# Supplementary material for: Combined Impact of Inflammation and Pharmacogenomic Variants on Voriconazole Trough Concentrations: A Meta-Analysis of Individual Data
Source: J Clin Med. 2021 May 13;10(10):2089. doi: 10.3390/jcm10102089 (PMC8152514; doi:10.3390/jcm10102089)
Supplement: Supplementary file 1 [file jcm-10-02089-s001.zip › jcm-1211686-supplementary.pdf]

Supplementary data

Table S1. Patient characteristics according to the studies included in the meta-analysis.

	Gautier-Veyret et al, 2015 [19]	Gautier-Veyret et al, 2019 [25]	Gautier-Veyret et al, 2020 [26]	Yamada et al, 2015 [30]	Lamoureux et al, 2015 [15]	Veringa et al, 2017 [28]	Total
Number of patients	28	57*	42	47	9	20	203
Number of VRC Cmin	255	62	150	47	21	219	754
Weight (kg)	67.0 (37.0-97.0)	68.0 (41.0-105)	71.0 (45.4-138)	52.0 (34.0-76.0)	56.0 (50.0-80.0)	71.3 (50.0-109)	68.0 (34.0-138.0)
Missing data	55 (21.6)	5 (8.1)	7 (4.7)	0 (0)	0 (0)	0 (0)	67 (8.9)
Main pathology:							
Hematological malignancies	28 (100)	57 (100)	42 (100)	27 (57.4)	-	15 (75)	169 (83.3)
Organ transplant	0 (0)	0 (0)	0 (0)	0 (0)	-	3 (15)	3 (1.5)
Other	0 (0)	0 (0)	0 (0)	20 (42.6)	-	2 (10)	22 (10.8)
Missing data	0 (0)	0 (0)	0 (0)	0 (0)	9 (100)	0 (0)	9 (4.4)
Daily dose (mg)	400 (52.7-800)	400 (200-800)	400 (200-700)	400 (200-600)	400 (200-500)	400 (200-1050)	400 (52.7-1050)
Route of administration							
IV	32 (12.5)	24 (38.7)	62 (41.3)	13 (27.7)	0 (0)	36 (16.4)	167 (22.1)
Oral	223 (87.4)	38 (61.3)	88 (58.7)	34 (72.3)	21 (100)	183 (83.6)	587 (77.9)
Liver function							
ASAT (UI/L)	33 (5-518)	22 (3-210)	26 (6-298)	27 (9-102)	27 (18-133)	30 (10-10360)	29.0 (3-10360)
Missing data	13 (5.1)	5 (8.1)	8 (5.3)	0 (0)	0 (0)	36 (16.4)	62 (8.2)
ALAT (UI/L)	46 (11-761)	29 (7-425)	27 (4-498)	19 (5-149)	26 (10-284)	38 (11-2605)	35.0 (4-8062)
Missing data	14 (5.5)	5 (8.1)	8 (5.3)	0 (0)	0 (0)	36 (16.4)	63 (8.4)
Concomitant PPI treatment	247 (96.8)	45 (72.6)	125 (83.3)	9 (19.1)	12 (57.1)	126 (57.5)	564 (74.8)
CYP2C19 phenotype							
EM	11 (39.3)	28 (49.1)	18 (42.8)	16 (34.0)	5 (55.6)	9 (45.0)	87 (42.9)
IM	7 (25.0)	13 (22.8)	12 (28.6)	25 (53.2)	1 (11.1)	6 (30.0)	64 (31.5)
PM	0 (0)	2 (3.5)	0 (0)	6 (12.8)	1 (11.1)	0 (0)	9 (4.4)
RM	8 (28.6)	14 (24.6)	12 (28.6)	0 (0)	2 (22.2)	4 (20.0)	40 (19.7)
UM	2 (7.1)	0 (0)	0 (0)	0 (0)	0 (0)	1 (5.0)	3 (1.5)
Missing data	0 (0)	0 (0)	0 (0)	0 (0)	0 (0)	0 (0)	0 (0)
CYP3A4 phenotype				ND		ND	
EM	21 (75)	47 (82.5)	39 (92.9)	-	7 (77.8)	-	114 (56.2)
IM	7 (25)	10 (17.5)	3 (7.1)	-	2 (22.2)	-	22 (10.8)
Missing data	0 (0)	0 (0)	0 (0)	47 (100)	0 (0)	20 (100)	67 (33.0)
CYP3A5 phenotype				ND		ND	
No expressor	26 (92.9)	49 (86.0)	33 (78.6)	-	8 (88.9)	-	116 (57.1)
Expressor	2 (7.1)	8 (14.0)	9 (21.4)	-	1 (11.1)	-	20 (9.9)
Missing data	0 (0)	0 (0)	0 (0)	47 (100)	0 (0)	20 (100)	67 (33.0)

ALAT = alanine aminotransferase; ASAT = Aspartate aminotransferase; Cmin = trough concentration; CYP = cytochrome P450; EM = extensive metabolizer; IM = intermediate metabolizer; IV = intravenous; PM = poor metabolizer; PPI = pomp proton inhibitor; RM = rapid metabolizer; UM = ultra-rapid metabolizer; VRC = voriconazole.

*Five patients were included in studies [19] and [25] with voriconazole trough concentrations determined at different times.

Table S2: Calculated combined genetic scores (number of patients) according to CYP2C19 and CYP3A4/5 genotypes.

			CYP2C19				
			*2/*2 or *2/*3	*2/*1 or *3/*1	*1/*1 or *2/*17	*17/*1	*17/*17
		Specific CYP genetic score	0	0.5	1	1.5	2
**CYP3A4/5**	3A4 *22/*1 and 3A5 *3/*3	0.5	0.5 (n=1)	1 (n=5)	1.5 (n=6)	2 (n=8)	2.5 (n=0)
	3A4 *1/*1 and 3A5 *3/*3 or 3A4 *22/*1 and 3A5 *3/*1	1	1 (n=2) ¶	1.5 (n=16) ¶¶	2 (n=55) #	2.5 (n=24) ##	3 (n=1)§
	3A4 *1/*1 and 3A5 *1/*3	1.5	1.5 (n=0)	2 (n=5)	2.5 (n=4)	3 (n=2)	3.5 (n=1)
	3A4 *1/*1 and 3A5 *1/*1	2	2 (n=0)	2.5 (n=1)	3 (n=3)	3.5 (n=2)	4 (n=0)

CYP = cytochrome P450. Light, medium, and dark grey indicate genetic scores < 2, = 2, and > 2, respectively.

¶ CYP3A4: *1/*1 (n = 2) and CYP3A5: *3/*3 (n = 2).

¶¶ CYP3A4: *1/*1 (n = 15), *22/*1 (n = 1) and CYP3A5: *3/*3 (n = 15); *3/*1 (n = 1).

CYP2C19: *1/*1 (n = 50), *2/*17 (n = 5), CYP3A4: *1/*1 (n = 54), *22/*1 (n = 1) and CYP3A5: *3/*3 (n = 54), *1/*3 (n = 1).

CYP3A4: *1/*1 (n = 24) and CYP3A5: *3/*3 (n = 24).

§ *CYP3A4: *1/*1* (n = 1) and *CYP3A5: *3/*3* (n = 1).

Table S3. Quality assessment of included cohort studies.

Study	Selection				Comparability	Outcome			Total score
	**Representativeness of the exposed cohort**	**Selection of the non-exposed cohort**	**Ascertainment of exposure**	**Outcome of interest not present at start of study**	**Comparability of cohorts on the basis of the design or analysis**	**Assessment of outcome**	**Was follow up long enough for outcomes to occur**	**Adequacy of follow up of cohorts**	
**Gautier-Veyret et al, 2015 [19]**	*	*	*	*	**	*	NA	NA	*******
**Gautier-Veyret et al, 2020 [26]**	*	*	*	*	**	*	NA	NA	*******
**Yamada et al, 2015 [29]**	*	*	*	*	**	*	NA	NA	*******
**Lamoureux et al, 2015 [15]**	*	*	*	*	**	*	NA	NA	*******
**Veringa et al, 2017 [28]**	*	*	*	*	**	*	NA	NA	*******

NA = not applicable.

Table S3bis. Quality assessment of included case control study.

Study	Selection				Comparability	Exposure			Total score
	Adequate definition of case	Representativeness of the cases	Selection of controls	Definition of controls	Comparability of cases on the basis of the design or analysis	Ascertainment of exposure	Same method of ascertainment for cases and controls	Non-response rate	
**Gautier-Veyret et al, 2019 [27]**	*	*	*	*	**	*	*	NA	********

NA = not applicable.
